# The moderating role of perceived health risks on the acceptance of genetically modified food

**DOI:** 10.3389/fpubh.2023.1275287

**Published:** 2024-01-25

**Authors:** Inna Cabelkova, Petra Sanova, Martin Hlavacek, David Broz, Lubos Smutka, Petr Prochazka

**Affiliations:** Czech University of Life Sciences Prague, Prague, Czechia

**Keywords:** environment, food, genetically modified food, health, nutritional profile, perceptions, policy, proteins

## Abstract

The public perspective on genetically modified foods (GMFs) has been intensely debated and scrutinized. Often, discussions surrounding GMF tend to revolve solely around the potential health risks associated with their consumption. However, it is essential to acknowledge that public perceptions of genetically modified foods are multifaceted, encompassing environmental concerns, ethical considerations, and economic implications. This paper studies the factors predicting GMF acceptance employing the representative sample of the Czech population (N = 884, aged 18–90 years, M ± SD: 48.17 ± 17.72; 53.40% women, 18.04% with higher education). The research relies on the Behavioral Change Model and the Health Belief Model. We employ hierarchical ordinal regressions to study the effects of information, environmental concerns, perceived health risks, food habits, purchasing habits, and socio-demographics on GMF acceptance. The results suggest that the (un)willingness to purchase GMF is primarily driven by the health risks - the environmental concerns were largely unimportant. The impact of information provision on GMF acceptance proved positive, suggesting information and education to be the main channels of creating public acceptance. The intrinsic interest regarding information related to GMF had an adverse impact on the perception of GMF morality. The benefits of the GMF proved unrelated to the GMF acceptance, indicating the gap in the information campaign. The research provides valuable insights for policymakers, public health professionals, and market researchers to communicate the GMF agenda effectively to the general public.

## 1 Introduction

Hunger, malnutrition, and the increase in population are currently the most urgent concerns. Presently, more than 820 million individuals lack access to sufficient food. In 2018, one out of every nine people on Earth faced malnutrition (1).

Genetically modified crops are suggested to offer an important contribution to address food shortages. They exhibit enhanced resistance to pests and diseases, as highlighted by research (2, 3). Furthermore, these crops can be genetically engineered to yield higher production and hold the promise of improved nutritional profiles, such as increased protein content (4, 5). They are also characterized by lower production costs (6, 7), greater adaptability to climate change (8, 9), and the potential to reduce food waste due to their extended shelf life (10–12). Additionally, these crops may offer enhanced taste and texture (10–12).

However, both the public and governments in Europa exhibit hesitance in embracing genetically modified foods (GMF). This reluctance is grounded in various factors, including concerns about potential health risks (13), apprehensions regarding environmental and biodiversity impacts (14), and ethical, moral, and religious considerations (15–17). Interestingly, this resistance persists despite the consensus among risk assessors and academics based on a plethora of risk assessment studies that GMFs are as safe for human and animal health as conventional crops (18). It’s noteworthy that the media often present a mixed view on GMF safety, where campaigns opposing GMFs frequently counter campaigns in support of them.

This paper aims to assess the power of environmental concerns, health risks, and information in predicting public acceptance of GMF, employing the representative sample of the Czech population (N = 884). The statistical model is constructed according to the principles of the Behavioral Change Model and the Health Belief Model. Methodologically, we rely on hierarchical ordinal regression analysis to assess predictors of willingness to buy GMF, taste GMF, and the perceived morality of GMF. We also perform principal component analysis to reduce the dimensionality of the indicators of environmental concern.

The paper is structured as follows. The first section discusses the main points of public discussion on GMF. The following sections are devoted to brief descriptions of Behavioral Change and Health Belief models. These models are employed to build the statistical model. Then, we describe the data, methods, and results. The final section provides the discussion and concludes.

## 2 The public discourse on GMF

Public discussions on genetically modified foods (GMFs) have been characterized by diverse and often conflicting opinions (19, 20). While some individuals and interest groups embrace GMFs as a potential solution to address global food security challenges, others express concerns about their possible health and environmental risks. Ethical considerations, corporate control of the food system, labeling requirements, and potential economic implications have also been central themes in public discussions on GMFs.

The type and purpose of modification stand out as the pivotal factors shaping attitudes toward various biotechnological methods (21, 22). As an illustration, a study on willingness-to-pay involving 713 participants (23) and 843 consumers (24) revealed the following preference order: (i) Organic; (ii) Cis- or transgenic with environmental benefits (pesticide-free crop cultivation); (iii) Conventional; (iv) Cisgenic; (v) Transgenic.

### 2.1 The health risks

Concerns regarding health risks have been a subject of scrutiny since the inception of genetically modified foods (GMFs) in the market (25–27). Among the foremost health-related apprehensions associated with GM foods, two significant areas are frequently highlighted: toxicity and allergenicity (13). To illustrate, shortly after the introduction of transgenic corn to the market, there were several instances of consumers reporting food allergy symptoms, such as headaches, diarrhea, nausea, and vomiting, which were attributed to the consumption of products containing GM corn (28, 29).

Another significant worry pertains to the potential alteration of our human DNA by recombinant DNA present in GM foods. This is envisaged to occur through the introduction of foreign genes into the human genome or cumulative modifications in our metabolic processes due to changes in dietary intake. However, the occurrence of horizontal gene transfer (HGT) across all organisms, is significantly lower in magnitude compared to gene transfer through sexual or asexual reproduction (30). Despite over two decades of widespread consumption of GM foods, there has been no substantiated instance of gene insertion into humans attributed to the consumption of GM foods (31).

### 2.2 Effects on environment

GMOs can have a range of potential effects on the environment. These include reduced biodiversity, outcrossing of genetically modified plants to non-modified or wild-relatives, disruption of natural ecosystems by the widespread introduction of GMOs, and reduced effectiveness of certain pest deterrents. Additionally, risks may be associated with the unintended transfer of genes between species that could lead to unpredictable effects on the environment and food webs (14). On the other hand, the positive effects of GM crops include the decreased use of herbicides, pesticides, and other chemicals for food production.

### 2.3 The ethical and moral aspects of GMF

Additional criticisms of genetically modified foods (GMF) revolve around moral and ethical considerations (15, 17, 32). From the ethical perspective genetically modified food (GMO) can be considered as ethically problematic due to its departure from the natural order of food production (16). GMO typically entails the modification of plant DNA to enhance its nutritional value or make it more resistant to diseases, pests, or environmental challenges. This manipulation of the natural food production process can be considered as a violation of fundamental principles of nature.

Moreover, concerns persist regarding potential long-term health risks stemming from the consumption of genetically modified foods and the unintended environmental consequences resulting from cross-pollination. Hence, many people regard genetically modified food as ethically questionable. Consequently, the challenge of gaining broad acceptance for GM foods stems not only from ethical principles but also from biological concerns associated with the complexities of the processes involved (7, 32).

In certain cultures, religious beliefs wield significant influence in determining what is deemed suitable for human consumption. Within these cultural contexts, genetically modified foods might clash with religious doctrines, thereby reducing their acceptance among the general population (33, 34). Many individuals who hold strong religious beliefs also express reservations about GMOs because they perceive these technologies as interfering with natural processes and disrupting the delicate balance of nature. There is apprehension that genetic manipulation may be inherently unpredictable or uncontrollable, while others view it as a form of “playing God,” supplanting a role that should solely belong to the divine.

Even among those who do not reject genetically modified foods based on religious convictions, some may abstain due to a deep reverence for the natural world or concerns about potential uncharted risks associated with their consumption. As GMOs continue to gain wider commercial use, discussions that intertwine ethics and belief are bound to become increasingly intertwined, influencing decisions that impact public policy and consumer choices.

### 2.4 The impact of information

While certain studies indicate that cultivating and manufacturing modified products can lead to reduced production expenses (6, 7), a substantial number of consumers opt for pricier non-GM alternatives based on principles rather than functional considerations. This aligns with the earlier noted limited public acceptance of GM foods, where consumers with high subjective knowledge but limited objective understanding exhibit the highest willingness to pay for non-GMO foods (35).

A substantial portion of the population is not well-informed about the scientific evidence that either supports or questions the use of GM technologies. The conflicting viewpoints presented by proponents and opponents of genetically modified foods (GMF) in media debates and the deliberate actions against GMF led by non-governmental organizations (NGOs) have contributed to a pervasive state of public uncertainty (36). Additionally, social networks disseminate information about the adverse effects of GMFs that may not always be rooted in scientific findings, further exacerbating this confusion (37). Interestingly, even individuals with limited knowledge about GMOs have the capacity to shape their children’s perceptions of GMOs (38).

Numerous research studies (39–41) have empirically established a direct connection between knowledge and attitudes, indicating that there is a direct and positive correlation between increasing familiarity with GM technologies and growing support for GM applications (42). Therefore, initiatives aimed at raising awareness can foster an informed public and a more objective assessment of the associated risks and benefits.

Nonetheless, it is important to recognize that the influence of knowledge is moderated by perceptions of the ethical implications of genetic modification rather than by political or religious affiliations (43). Some studies have cast doubt on the direct association between scientific knowledge and attitudes, revealing that the link between science-based information on GMF and the formation of accurate public perceptions is often weak and, in certain cases, non-existent (44). Public acceptance can also be influenced by government regulatory policies and laws concerning the cultivation and sale of genetically modified products, as individuals who disagree with such policies may protest against these products, even if they are not directly involved.

### 2.5 The role of the state

To alleviate the perceived risks mentioned above, it is possible to partially address them by fostering trust in regulatory bodies, scientists, industry, and by building public confidence in government and corporations (45–47). Nevertheless, a significant portion of the population remains skeptical about large corporations that dominate the production of genetically modified foods, perceiving their motivations as primarily profit-driven rather than driven by safety or health benefits. Companies like Monsanto, often cited as emblematic of the industry’s supposed shortcomings (48, 49), are frequently mentioned in this context.

This skepticism can lead to doubts regarding the safety of genetically modified foods. As a result, consumers commonly express concerns about potential changes in food quality, unequal competition between GMO and non-GMO suppliers, biopiracy, and related issues (27).

### 2.6 The aim of the paper and hypotheses

Following the public discussion above, this paper aims to assess the role of health risks, environmental concerns, and information in predicting the public acceptance of GMF. Central hypotheses are formulated as follows:

*H1*. GMF acceptance is predicted by environmental concerns.

*H2*. GMF acceptance is negatively predicted by perceived health risks.

*H3*. GMF acceptance is predicted by the availability of relevant information and interest in the subject.

## 3 Materials and methods

### 3.1 The health belief model and behavioral change model

The Health Belief Model presents four major constructs that govern people’s behavior related to health outcomes: perceived susceptibility, perceived severity, perceived benefits, and perceived barriers (50). The impact of these constructs on health-related action is then modified by socio-economic conditions (such as age, gender, education, personality, and standard of living) and knowledge of the subject. In our case, the perceived susceptibility and severity are approximated by the current state of health and the beliefs on the impact of GMF on health. The benefits are related to the importance of various aspects of food purchasing and consumption, such as price, ingredients, frequency of food purchasing, the importance of self-catering, etc. (see the indicators presented in the next sections). We suggest that the introduction of GMF lowers the price (51), and properties of GMF may make food consumption easier (for example, through longer shelf life and easier storage, which will lower the need for frequent food purchasing (52)). The effects of information are then controlled by the indicators of information on GMF. Socio-economic and personality effects are approximated by age, gender, education, town size, standard of living, life satisfaction, and belief in God.

The behavioral change model presents a more general idea of the factors impacting behavioral outcomes. Here, the behavior is predicted by knowledge, awareness and attitudes, and socio-demographics (53, 54). In our case, the possible environmental outcomes of GMF production and environmental concerns. We combine both approaches to construct the following model:

### 3.2 The method

**Graph 1:**
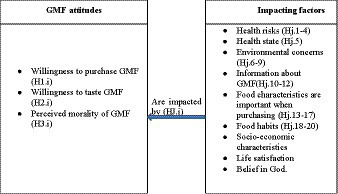
The model and hypotheses (Hj.i).

We apply hierarchical ordinal regression analysis to test the hypotheses presented in Graph 1. The hierarchical part of ordinal regression analysis included two steps. First, we tested the model with all the explanatory variables according to formula (1).


(1) $$GMF\;\mathrm{Attitudes}=\mathrm{Logit}\;\left(\begin{array}{l} a_{0}+a_{1-3}\;\mathrm{Information}+\\ a_{4-8}\;\mathrm{Health}+\\ a_{1-12}\;\mathrm{Environment}+\\ a_{13-17}\;\mathrm{Food}\;\mathrm{Purchasing}+\\ a_{18-20}\;\mathrm{Food}\;\mathrm{habits}+\\ a_{21-27}\;\mathrm{Socio}-\mathrm{demographics}+e \end{array}\right)$$

In the second stage, we excluded the group of variables related to health effects and computed ordinal regression according to the following formula (2):


(2) $$GMF\;\mathrm{Attitudes}=\mathrm{Logit}\;\left(\begin{array}{l} a_{0}+a_{1-3}\;\mathrm{Information}+\\ a_{9-12}\;\mathrm{Environment}+\\ a_{13-17}\;\mathrm{Food}\;\mathrm{Purchasing}+\\ a_{18-20}\;\mathrm{Food}\;\mathrm{habits}+\\ a_{21-27}\;\mathrm{Socio}-\mathrm{demographics}+e \end{array}\right)$$

Where

*GMF Attitudes -* Would buy food with a GM ingredient, Would taste approved GMF, Genetically modifying crops are morally unacceptable.

*Information -* Have heard about genetically modified crops, Interested in GMF, Have enough information about GMF.

*Health -* Own state of health, Consuming GMF is safe, The health effects of GMF are sufficiently researched, Consuming GMF can change human DNA, GMF can endanger human health.

*Environment - the* importance of the impact of food production on the environment, reduces waste, saves resources to protect the environment, recycles.

*Food Purchasing –* the importance of origin, package material, price, ingredients, and package size.

*Food habits* - Food consumption is important, Frequency of food purchasing, Number of meals per day.

*Socio-demographics –* Gender, Age, Education, Town size, Household standard of living, Life satisfaction, Belief in God.

The description of the variables above is presented in the section Indicators.

We compared the pseudo-R-square of both models and inferred the moderation effects of the excluded variables.

### 3.3 The data

The data were collected in July 2021 in a survey entitled Food 2021 (18) conducted by the Czech Institute of Sociology. A total of 884 respondents representing the population of the Czech Republic (aged 18–90 years, M ± SD: 48.17 ± 17.72; 53.40% women, 18.04% with higher education) answered the questions in the questionnaire voluntarily and anonymously under the supervision of 139 experienced interviewers (combination of PAPI (68%) and CAPI (32%) interviews). As the quality of the filled-out questionnaires was considered very good, all the questionnaires were included in the data sample. All participants were Czech native speakers living in the Czech Republic. Respondents were selected by quota sampling. Quota features were Region (NUTS 3), size of place of residence, gender, age, and education. The data sample is representative of the Czech Republic. The data were kindly provided by the Czech Social Science Data Archive (55).

### 3.4 Indicators

#### 3.4.1 GMF acceptance

The GMF acceptance is studied in three aspects – the willingness to purchase GMF, the willingness to try GMF, and the moral acceptability of GMF. The relevant questions in the questionnaire were formulated as follows:

*“To what extent do you agree or disagree with the following statements?*

- *If you discovered that you had a food item in your shopping cart containing an ingredient from genetically modified crops, you would still buy it.*
- *Genetically modifying crops is morally unacceptable.*
- *If you had the chance, would you taste an approved and verified food from genetically modified crops?”* (18)

Table 1 shows that the fear of the population of GMF is rather mild – 62% of the respondents are willing to taste GMF. However, the willingness to buy is rather small - only 35% of the respondents would buy GMF. The biggest ambiguity concerned the perceived moral acceptance of the GMF – 52% of the respondents did not have an opinion or were undecided.

**Table 1:** Indicators of attitudes for GMF.

Question	Definitely agree	Rather agree	Undecided	Rather disagree	Definitely disagree	No opinion
Would buy food with a GM ingredient	8.3	27.1	21.3	16.1	10.7	16.6
	Definitely yes	Rather yes	Rather no	Definitely no	No opinion	
Would taste approved GMF	10.9	13.5	30.4	15.4	7.5	22.3
GMFs are morally unacceptable	3.1	10.9	25.7	60.4		

The distribution of the respondents (%).

Given the relatively high number of people with no opinion about the perception questions, we added these people to the group of people “undecided” where possible (this is category 3 on the 5-point Likert scale).

#### 3.4.2 Information about GMF

Information is essential for opinion creation. In this study, we employ indicators for the availability and sufficiency of this information, and we also control the level of interest in the topics. The indicators, the scales, and the distribution of the respondents are presented in Table 2.

**Table 2:** Indicators of GMF information.

Have heard about GMF	No	Yes, but does not know what it refers to	Yes, and roughly knows what it involves	Yes, and knows well what it involves	
	27.7	31.9	33.4	6.8	
Interested in GMF	Definitely yes	Rather yes	Rather no	No	Does not know
	3.3	12.7	32.9	48.4	2.6
Have enough information about GMF	Definitely enough	Rather enough	Rather not enough	Definitely not enough	Does not know
	3.1	14.1	32.5	40.8	9.4

The respondents who answered “do not know” were excluded from further analysis.

#### 3.4.3 Perceived GMF effects on health

The literature suggests that the perceived effects on health are one of the most important informational problems affecting legislation and public use of GMF. Table 3 summarizes the indicators used in this paper.

**Table 3:** Indicators of perceived GMF effects on health.

Own state of health, assessment	Very good	Good	Average	Bad	Very bad	
	20.00	42.30	29.30	7.50	0.90	
Consuming GMF is safe	Definitely agree	Rather agree	Undecided	Rather disagree	Definitely disagree	No opinion
	4.30	18.40	26.80	16.20	8.90	25.10
The health effects of GMF are sufficiently researched.	Definitely agree	Rather agree	Undecided	Rather disagree	Definitely disagree	No opinion
	5.90	22.50	21.60	15.50	7.90	26.50
Consuming GMF can change human DNA	Definitely yes	Rather yes	Rather no	Definitely no	No opinion	
	5.40	15.70	21.20	21.40	36.20	
GMF can endanger human health	Definitely yes	Rather yes	Rather no	Definitely no	No opinion	
	10.30	24.70	24.40	7.00	33.60	

The distribution of the respondents (%).

The respondents with no opinion were joined with the group Undecided for further analysis.

#### 3.4.4 Environmental concerns

The first indicator of environmental concerns studied the subjective level of importance of the impact of food production on the environment (definitely important, 11.10% of the respondents; rather important, 37.30%; rather unimportant, 30.0%; definitely unimportant, 11.00%; and no opinion 3.70%).

Next, we mapped environmental concerns by the frequency of engaging in environmentally friendly behavior (Table 4).

**Table 4:** Indicators of environmental concerns.

How often does the respondent	Never	Rarely	Sometimes	Often	Always	No opinion
Use own reusable shopping bag	5.50	8.00	16.20	24.20	44.60	1.40
Use reusable bags for purchasing fruits and vegetables	41.30	13.90	17.30	12.90	10.70	3.70
Use reusable bottles for drinks	33.30	11.80	22.90	17.80	13.10	1.10
Use environmentally friendly detergents	17.90	18.60	28.70	17.90	6.20	10.60
Prefer purchasing Czech-made foods	7.70	11.80	31.40	32.80	13.10	3.10
Pack the food into reusable boxes	28.80	17.20	21.00	20.20	10.40	2.10
Avoid single-use plastic products	13.70	15.40	23.80	25.10	19.70	2.30
Limit car trips to protect the environment	40.20	23.40	17.80	9.60	4.20	4.50
Save energy and water to protect the environment	17.40	19.00	27.80	22.40	11.80	1.50
Sort waste	4.60	6.80	15.80	30.90	41.10	0.70
Compost	45.10	6.40	13.10	14.90	18.70	1.60

The distribution of the respondents (%).

*N* = 727. The respondents with No opinion were excluded from further analysis.

To reduce the dimensionality of the model, we applied the Principal Component Analysis (PCA) to the indicators presented in Table 4 and used the regression-based factor scores in further analysis. The results of PCA are reported in the Data transformation section. Three components were extracted: the tendency to reduce and sort waste, save resources, and for recycling.

#### 3.4.5 The importance of food characteristics when purchasing

Consumers consider a number of characteristics when purchasing their food to various extents. They take into account the ingredients, package material and size, origin, and, obviously, the price (Table 5). We hypothesize that these factors are also important predictors of attitudes to GMF.

**Table 5:** Indicators of the importance of food characteristics when purchasing.

	Mean	Std. deviation
Origin	3.1	1.479
Package material	4.89	1.298
Price	2.22	1.438
Ingredients	2.78	1.412
Package size	3.48	1.472

*N* = 799, Min = 1 (very important), Max = 6 (least important).

#### 3.4.6 The importance of food and food habits

The indicators of the subjective importance of food and food habits are presented in Table 6.

**Table 6:** Indicators of the importance of food and food habits.

Food consumption important	Definitely important	Rather important	Rather unimportant	Definitely unimportant		
	43.30	43.00	9.80	3.40		
Frequency of food purchasing	Daily	Several times a week	1x a week	1x per 14 days	Less than 1x per 14 days	No answer
	9.80	50.80	23.50	5.50	3.50	6.70
Number of meals per day	One meal	Two meals	Three meals	Four meals	Five meals	More than five
	0.10	8.50	39.90	30.70	16.40	4.10

The distribution of the respondents (%).

#### 3.4.7 Socio-economic characteristics of the respondents and other

We control for gender, age, and education (age 18–90 years, M ± SD: 48.17 ± 17.72; 53.40% women, 18.04% with higher education), subjective town size (from a big city to small village), the household standard of living (very good, 13,l2%; rather good, 45.5%; neither good nor bad, 33.9%; rather bad, 6.4%; very bad, 0.9%), life satisfaction (very satisfied 20.8%; rather satisfied, 50%; neither satisfied nor dissatisfied, 21.3%; rather dissatisfied, 5.7%; very dissatisfied 1.2%), and belief in God (69.9% non-believers).

### 3.5 Data transformations and treatment of missing values

As the level of information on GMF is still low, some questions exhibited high numbers of respondents with no opinions. As stated in the previous chapters, we joined the respondents with No opinion with the group Undecided. This data transformation forms one of the limitations of the study. In case there was no option “undecided,” the respondents with No opinion were excluded from further analysis.

#### 3.5.1 Data transformations. Environmental concern. The principal component analysis

In order to reduce the dimensionality of the model, we applied the Principal Component analysis to the set of variables representing the respondents’ actions to protect the environment (Indicators of environmental concerns, Table 4). An Eigenvalue of 1 or higher determined factor extraction and all variables were extracted as expected. The Bartlett test of sphericity with a Chi-Square value of 1716.968 (*p* < 0.001) and Kaiser-Meyer-Olkin Measure of sampling adequacy was equal to 0.852 (>0.8) suggests that the data are suitable to identify factor dimensions. The two factors extracted cumulatively explain 54.095% of the total variance. The rotated component matrix is presented in Table 7.

**Table 7:** The rotated component matrix for components describing indicators of environmental concern.

	Question:	Component		
	How often does the respondent	1	2	3
Reduce waste	Sort waste	0.765	0.059	0.179
	Use own reusable shopping bag	0.623	0.138	0.055
	Prefer purchasing Czech-made foods	0.614	0.326	0.125
Saving resources to protect the environment	Limit car trips to protect the environment	−0.062	0.842	0.113
	Save energy and water to protect the environment	0.394	0.637	0.136
	Avoid single-use plastic products	0.457	0.545	0.101
	Use environmentally friendly detergents	0.410	0.523	0.227
Recycling	Use own reusable bottle for drinks	0.018	0.127	0.796
	Pack the food into reusable boxes	0.177	0.155	0.760
	Compost	0.421	−0.078	0.500
	Use reusable bags for purchasing fruits and vegetables	0.105	0.367	0.495
% of variance explained		34.25	10.593	9.252

Extraction method: principal component analysis. Rotation method: varimax with Kaiser Normalization.

The regression-based factor scores for all three components were saved as variables and used for further analysis.

## 4 Results and discussion

### 4.1 Results

The results of the first stage of hierarchical ordinal regression analyses are presented in Table 8 below (formula 1), and the second stage in Table 9 (formula 2).

**Table 8:** Factors predicting GMF consumption.

	Purchase GMF		Try GMF		GMF immoral	
	Estimate	Sig.	Estimate	Sig.	Estimate	Sig.
Threshold = 1	−1.925	0.201	1.488	0.337	−0.732	0.633
Threshold = 2	1.05	0.483	4.371*	0.005	0.673	0.66
Threshold = 3	3.268*	0.03	5.820***	<0.001	3.785*	0.014
Threshold = 4	5.198***	<0.001			5.589***	<0.001
*Information about GMF*						
Heard of GMF	−0.249*	**0.031**	−0.199	0.097	0.266*	**0.025**
Interested in GMF	−0.206	0.095	0.081	0.523	0.284*	**0.024**
Enough Info about GMF	0.038	0.643	0.099	0.24	0.033	0.691
*GMF effects on health*						
State of own Health	−0.135	0.272	−0.143	0.268	−0.047	0.708
GMF is safe	1.078***	**<0.001**	0.778***	**<0.001**	−0.753***	**<0.001**
The effects of GMF on health are scientifically investigated	0.610***	**<0.001**	0.17	0.124	−0.233*	0.033
Consuming GMF can change DNA	−0.254**	**0.001**	−0.154	0.055	0.346***	**<0.001**
GMF can endanger his health	−0.457***	**<0.001**	−0.316***	**<0.001**	0.563***	**<0.001**
*Environmental concerns*						
Effect of food production on environment important	−0.202	0.083	−0.260*	**0.033**	0.099	0.405
Reduce Waste (component1)	0.038	0.695	−0.293**	**0.005**	−0.133	0.182
Save resources (component 2)	−0.035	0.691	0.06	0.525	−0.048	0.598
Recycling (component 3)	0.084	0.337	0.072	0.424	0.055	0.532
*Aspects of food important when purchasing*						
Origin	0.028	0.724	0.036	0.664	0.087	0.29
Packaging	−0.137	0.067	−0.089	0.253	0.173*	**0.024**
Price	−0.024	0.746	0.06	0.43	0.052	0.487
Ingredients	0.075	0.379	0.028	0.751	0.121	0.166
Package size	0.095	0.208	0.071	0.361	0.012	0.874
*Food habits*						
Number of meals per day	−0.023	0.784	0.103	0.245	−0.1	0.245
Importance of self-catering	0.042	0.727	0.189	0.136	−0.134	0.27
Frequency of food purchasing	0.107	0.269	−0.084	0.414	0.097	0.323
*Socio-demographics*						
Gender	0.304	0.071	0.05	0.779	−0.01	0.955
Age	0.008	0.133	0.006	0.3	−0.002	0.785
Education	0.133	0.161	0.036	0.71	−0.04	0.676
Town size	0.002	0.97	0.187**	**0.001**	−0.044	0.432
Household standard of living	−0.056	0.65	0.047	0.714	0.09	0.473
Life satisfaction	−0.064	0.617	0.274*	**0.044**	−0.18	0.171
Non believer in God	0.063	0.733	0.322	0.102	−0.179	0.345
*Model fitting information, Sig.*						
Sig.		<0.001		<0.001		<0.001
*N*	625		586		622	
*Pseudo R-Square*						
Cox and Snell	0.51		0.323		0.394	
Nagelkerke	0.538		0.352		0.422	
McFadden	0.242		0.156		0.185	

Results of ordinal regression analysis (formula 1).

Link function: Logit. ***significant on 0.1% level. **significant on 1% level, *significant on 5% level. Components 1, 2, 3 denote the three components of PCA presented in Table 7.

**Table 9:** Factors predicting attitudes to GMF.

	Purchase GMF		Try GMF		GMF immoral	
	Estimate	Sig.	Estimate	Sig.	Estimate	Sig.
Threshold = 1	−2.515	0.059	0.335	0.811	−1.012	0.489
Threshold = 2	−0.492	0.711	2.665	0.059	0.327	0.823
Threshold = 3	0.984	0.459	3.841**	0.007	2.191	0.135
Threshold = 4	2.223	0.095			3.376*	0.023
*Information about GMF*						
Heard of GMF	−0.082	0.443	−0.106	0.354	−0.637***	**<0.001**
Interested in GMF	−0.029	0.801	0.158	0.19	−0.116	0.361
Enough Info about GMF	0.053	0.483	0.088	0.278	−0.001	0.99
*Environmental concerns*						
Effect on environment important	−0.071	0.516	−0.214	0.065	−0.061	0.608
Reduce Waste (component 1)	−0.031	0.731	−0.305**	**0.002**	−0.515***	**<0.001**
Save Resources (component 2)	0.012	0.885	0.056	0.531	0.175	0.055
Recycling (component 3)	0.121	0.133	0.081	0.344	0.209*	**0.021**
*Importance when purchasing*						
Origin	−0.081	0.283	−0.036	0.653	0.183*	**0.027**
Packaging	−0.09	0.203	−0.086	0.254	−0.118	0.122
Price	−0.014	0.837	0.067	0.356	−0.131	0.096
Ingredients	0.028	0.725	0.019	0.824	0.034	0.702
Package size	0.025	0.722	0.047	0.529	0.152	0.053
*Food habits*						
Number of meals per day	0.022	0.78	0.099	0.247	−0.012	0.889
Importance of self-catering	0.026	0.814	0.121	0.318	0.043	0.726
Frequency of food purchasing	0.159	0.08	−0.02	0.838	−0.056	0.574
*Socio-demographics*						
Gender (men)	−0.059	0.709	−0.186	0.273	−0.033	0.849
Age	0.01	0.059	0.007	0.195	0.012*	**0.036**
Education	0.143	0.108	0.053	0.571	0.042	0.669
Town size	0.002	0.969	0.176**	**0.002**	−0.001	0.981
Household standard of living	−0.028	0.81	−0.002	0.986	−0.078	0.544
State of own Health	−0.066	0.566	−0.068	0.581	0.088	0.488
Life satisfaction	0.012	0.919	−0.143	0.438	−0.154	0.254
Non believer in God	−0.401	0.02	0.291*	**0.025**	0.375	0.054
*Model fitting information*						
Sig.		0.222		<0.001		<0.001
*N*	624		588		626	
*Pseudo R-Square*						
Cox and Snell	0.043		0.09		0.192	
Nagelkerke	0.046		0.098		0.212	
McFadden	0.015		0.038		0.091	

Results of ordinal regression analysis without health risks (Formula 2).

Link function: Logit. ***significant on 0.1% level. **significant on 1% level, *significant on 5% level. Components 1, 2, 3 denote the three components of PCA presented in Table 7.

The bulleted results of Table 8 are presented in Appendix A1 in Supplementary material. Here, we will summarize the findings.

Table 8 suggests that the health risks significantly impact GMF attitudes. The indicators of perceived health risks negatively predicted the willingness to try and purchase GMF and the perceived morality of GMF. The indicators of health risk were unrelated to checking GMO content at the purchase stage.

Environmental concerns and practices were unrelated to the willingness to try or purchase GMF, the knowledge of real GMO content in the food he eats, and the perceived morality of GMF (see Appendix A1 in Supplementary material).

The role of information showed positive - the level of personal knowledge of GMF and the persuasion that GMF is sufficiently investigated positively predicted willingness to purchase GMF; the level of personal knowledge of GMF was associated with higher moral acceptability of GMF. On the other hand, the interest in GMF negatively predicted the perceived morality of GMF.

Contrary to the idea that the respondents might appreciate the benefits of GMF in terms of lower price or higher shelf life (the latter enabling them to shop less often), these variables did not appear significant in the abovementioned analysis. Surprisingly, the results suggest that the more important the packaging, the more he considers GM food immoral.

Socio-demographic, economic, psychological, and religious characteristics were not significantly related to GMF attitudes, with two exceptions: town size and life satisfaction are positively related to the willingness to try GMF.

In order to test the predictive (and moderating) power of the perceived health effects of GMF as opposed to other variables, we conducted the second stage of the ordinal regression analyses, where all the variables representing the health effects were excluded from the analysis. The results are presented in Table 9.

The results suggest that excluding the variables representing the health effects of the GMF led to significant changes in the predictive power of the models for willingness to purchase GMF. While the original Pseudo R2 ranged from 24 to 51% (depending on the indicator of pseudo R2) and the original models were statistically significant at 0.1% level, the exclusion of health variables led to a reduction of Pseudo R2 to the level of 1–5% and to the loss of statistical significance of the whole model in the case of willingness to purchase. Thus, the results indicate that health effects can be considered the most powerful predictors of the willingness to purchase GMF.

The effect of the exclusion of health variables on the other two regressions was less pronounced as the regressions stayed statistically significant on 0.1% level. However, the variability explained by the model as measured by Pseudo R2 decreased considerably.

The moderating effect of the health risks manifested itself only in the case of one information variable (heard of GMF) when the association before exclusion was positive (more information about GMF positively predicted the perception that GMF is immoral), while after the same association proved to be negative. In addition, some environmental concerns got statistical significance after the exclusion of health effects.

### 4.2 Discussion

The findings presented above underscore several crucial points. Firstly, the absence of statistical significance regarding price and food habits suggests that consumers may not yet be fully aware of the potential benefits of GMF. This is comprehensible given that GMF is subject to extensive regulations in many countries, making consumers believe, that the GMF is controversial and diverting their attention from its cost-effectiveness. While the broader public discourse frequently emphasizes GMF’s potential to address global food security, its individual contributions to enhancing consumers’ lifestyles in developed nations are less conspicuous.

On a more positive note, the impact of information on the willingness to try and purchase GMF is affirmatively supported. This implies that information dissemination plays a pivotal role in boosting public acceptance of GMF. The significance of credible, scientifically-backed communication and education from authoritative sources cannot be overstated (42, 56, 57). Consequently, these communication and educational strategies should be a focal point when devising public education initiatives (58).

It’s also worth considering that these communication and education strategies should target address individuals who are notably interested in GMF but perceive them as morally objectionable (the association between interest in GMF and the perceived immorality of GMF is confirmed as positive). The role of opinion polarization on social networks needs further exploration in this context, as it’s plausible that heightened interest in the topic leads these individuals to join discussion groups that view GMF as ethically problematic. The availability of information on GMO is intrinsically related to precise labeling, as the labels themselves may provide up-to-date information on GMO products. The detailed labeling and trust in the European approval processes proved to be significant predictors of the public attitude to GMO (45–47, 57).

Notably, health-related concerns played a more significant role in the willingness to purchase GMF as opposed to the willingness to try them. Concerns about the perceived lack of safety of GMF and the assumed health risks associated with them had a negative impact on both the willingness to purchase and the willingness to try these products. However, the notion that consuming GMF could result in alterations to human DNA and the perception of limited scientific knowledge about GMF had a negative influence on the willingness to purchase but did not significantly affect the willingness to try GMF.

It is quite concerning that as much as one-fifth of the sample believes that consuming GMF can lead to changes in human DNA. If we include respondents with no opinion, this percentage increases to 56%. These individuals collectively constitute a receptive audience for various forms of misinformation that can originate from sources in both social and traditional media. Similarly, the perception that the health effects of GMF are insufficiently researched is largely speculative and is likely being promoted in the mass media (59).

The ethical and moral aspects of GMF are often deliberated through a religious lens (33, 34). Nonetheless, in our specific case, perceived morality did not exhibit any association with the belief in God. It could be inferred that in the context of the Czech Republic, the ethical dimension is more closely related to potential environmental and health impacts of GMF (as seen in research such as (28, 29)) and the overall intricacies of the biological processes involved (similar to the findings of (7, 32)).

## 5 Conclusion

Genetically modified foods (GMF) represent a potential solution to the growing global food demand, offering a way to feed the increasing world population. However, concerns persist about the environmental and health implications of GMF, prompting the need for further research. Governments and the general public remain cautious about widespread adoption of GMF, which limits the realization of their full potential benefits.

This paper studied the impacts of perceived health risks of GMF, environmental concerns, and information about GMF on the GMF acceptance represented three indicators: willingness to try and taste GMF and the perceived morality of GMF. We also studied the importance of possible positive effects of the GMF, such as lower price or larger shelf life. The research was guided by the Behavioral Change Model (BCM) and the Health Belief Model (HBM) to understand the factors that shape acceptance of GMF. We employed the representative data of the Czech population (N = 884, aged 18–90 years, M ± SD: 48.17 ± 17.72; 53.40% women, 18.04% with higher education) to test the model and hypotheses. Expectedly, the health risks proved to be the most important predictor of the willingness to purchase GMF. The impact of health risks on willingness to try was less pronounced. The environmental risks of GMF, as related to the environmental concerns and actions of the population, were largely unimportant.

The impact of information proved positive, suggesting information and education to be the main channel of creating public acceptance. The information campaign needs to explain not only the benefits of GMF on a worldwide scale but also the benefits to the particular consumer in terms of lower prices. According to the Health Belief Model, this may partially compensate for the GMF risks. The opinion polarization present primarily (but not only) on social networks also needs to be considered, as our results suggest that the interest in GMF predicts the perceived immorality of GMF. We suggest that interested individuals might share these opinions in the online and offline discussion forums.

The findings above reaffirmed the Czech Republic’s consistent positive public perception of genetically modified food (GMF) over time, aligning it with countries like Spain, Portugal, and Malta (refer to Daye et al. (60). In the Czech Republic, there was a notable willingness to taste GMF, with 62% of respondents expressing their readiness to do so. Moral concerns were relatively low, as only 15% of respondents considered GMF immoral. Hence, it is cautiously suggested that the results presented in this paper may be applicable to other European countries with similarly favorable perceptions of GMF, specifically Spain, Portugal, and Malta. However, the applicability of the findings is contingent upon the type of genetic modification, as public perception proved to be highly sensitive to this factor (60).

These results underscore the significance of incorporating health risks into the assessment of individuals’ willingness to embrace GMF, underlining the necessity for tailored communication strategies that can effectively tackle health-related apprehensions. This research yields valuable insights for policymakers, public health experts, and market researchers to efficiently convey the advantages of GMF while mitigating concerns tied to health, thus boosting its acceptance among consumers.

## Data availability statement

Publicly available datasets were analyzed in this study. This data can be found here: https://archiv.soc.cas.cz/en/pristup-k-datum-2/nase-data upon registration. Other raw data used in this study cannot be published due to restrictions given by the data collecting agency-inquiries about this data can be directed to the corresponding author.

## Ethics statement

The studies involving humans were approved by Ethics Committee of the Czech University of Life Sciences. The studies were conducted in accordance with the local legislation and institutional requirements. The participants provided their written informed consent to participate in this study.

## Author contributions

IC: Conceptualization, Formal analysis, Methodology, Supervision, Writing – original draft. PS: Formal analysis, Investigation, Writing – review & editing. MH: Data curation, Formal analysis, Investigation, Writing – original draft, Writing – review & editing. DB: Formal analysis, Investigation, Writing – review & editing. LS: Funding acquisition, Investigation, Supervision, Investigation. PP: Funding acquisition, Investigation, Validation, Investigation.

## References

[1] Verma V Negi S Kumar P Srivastava DK. (2021). Global status of genetically modified crops. In: Kumar Srivastava D Kumar Thakur A Kumar P. editors. Agric Biotechnol Lat Res Trends. Singapore: Springer.

[2] Talakayala A Sumalatha K Mallikarjuna G. Genetic engineering of crops for insect resistance: an overview. J Biosci. (2020) 45:114. doi: 10.1007/s12038-020-00081-y 33051408

[3] Yali W. Application of genetically modified organism (GMO) crop technology and its implications in modern agriculture. Int J Appl Agric Sci. (2022) 8:14–20. doi: 10.17352/2455-815X.000139

[4] Gbashi S Adebo O Adebiyi A Targuma S Tebele S Areo OM. Food safety, food security and genetically modified organisms in Africa: a current perspective. Biotechnol Genet Eng Rev. (2021) 37:30–63. doi: 10.1080/02648725.2021.1940735, PMID: 34309495

[5] Vega Rodríguez A Rodríguez-Oramas C Sanjuán Velázquez E Hardisson de la Torre A Rubio Armendáriz C Carrascosa Iruzubieta C. Myths and realities about genetically modified food: A risk-benefit analysis. Appl Sci. (2022) 12:2861. doi: 10.3390/app12062861

[6] Azadi H Ho P. Genetically modified and organic crops in developing countries: a review of options for food security. Biotechnol Adv. (2010) 28:160–8. doi: 10.1016/j.biotechadv.2009.11.003 19913085

[7] Ekici K Sancak YC. A perspective on genetically modified food crops. Afr J Agric Res. (2011) 6:1639–42.

[8] Garland S. EU policy must change to reflect the potential of gene editing for addressing climate change. Glob Food Sec. (2021) 28:100496. doi: 10.1016/j.gfs.2021.100496

[9] Zaidi A Vanderschuren H Qaim M Mahfouz MM Kohli A Mansoor S. New plant breeding technologies for food security science. Science. (2019) 363:1390–1. doi: 10.1126/science.aav6316 30923209

[10] Asrey R Barman K Prajapati U Sharma S Yadav A. Genetically modified fruit and vegetable-an overview on senescence regulation, postharvest nutraceutical quality preservation and shelf life extension. J Hortic Sci Biotechnol. (2021) 96:271–87. doi: 10.1080/14620316.2020.1845986

[11] Islam R. Assessment of the effects of genetically modified (GM) foods: a brief study on health and environmental concerns. J Mater Environ Sci. (2020) 11:1676–88.

[12] Kamthan A Chaudhuri AB Kamthan M Datta A. Genetically modified (GM) crops: milestones and new advances in crop improvement. Theor Appl Genet. (2016) 129:1639–55. doi: 10.1007/s00122-016-2747-6 27381849

[13] Zhang C Robert W Han Z. Genetically modified foods: a critical review of their promise and problems. Food Sci Human Wellness. (2016) 5:116–23. doi: 10.1016/j.fshw.2016.04.002

[14] Tsatsakis A Nawaz MA Tutelyan VA Golokhvast KS Kalantzi OI Chung DH. Impact on environment, ecosystem, diversity and health from culturing and using GMOs as feed and food. Food Chem Toxicol. (2017) 107:108–21. doi: 10.1016/j.fct.2017.06.033, PMID: 28645870

[15] Green N. An analysis of some ethical argumentation about genetically modified food. Argu Comput. (2023):1–20. doi: 10.3233/AAC-220014

[16] Knight A. Perceptions, knowledge and ethical concerns with GM foods and the GM process. Public Underst Sci. (2009) 18:177–88. doi: 10.1177/0963662507079375 19579682

[17] Kumar N Sonia Y. Review on genetically modified organism foods. J Pharm Res Int. (2021) 33:2815–23. doi: 10.9734/jpri/2021/v33i60B34946

[18] Smyth SJ McHughen A Entine J Kershen D Ramage C Parrot W. Removing politics from innovations that improve food security. Transgenic Res. (2021) 30:601–12. doi: 10.1007/s11248-021-00261-y 34053007 PMC8164681

[19] Batista R Oliveira MM. Facts and fiction of genetically engineered food. Trends Biotechnol. (2009) 27:277–86. doi: 10.1016/j.tibtech.2009.01.005 19324440

[20] Kumar K Gambhir G Dass AB Tripathi K Singh A. Genetically modified crops: current status and future prospects. Planta. (2020) 251:1–27. doi: 10.1007/s00425-020-03372-8 32236850

[21] Spök A Sprink T Allan AC Yamaguchi T Dayé C. Towards social acceptability of genome-edited plants in industrialised countries? Emerging evidence from Europe, United States, Canada, Australia, New Zealand, and Japan. Front Genome Edit. (2022) 4:899331. doi: 10.3389/fgeed.2022.899331 PMC9473316 36120531

[22] Strobbe S Wesana J Van Der Straeten D De Steur H. Public acceptance and stakeholder views of gene edited foods: a global overview. Trends Biotechnol. (2023) 41:736–40. doi: 10.1016/j.tibtech.2022.12.011 36658005

[23] Edenbrandt AK. Demand for pesticide-free, cisgenic food? Exploring differences between consumers of organic and conventional food. Br Food J. (2018) 120:1666–79. doi: 10.1108/BFJ-09-2017-0527

[24] Edenbrandt AK Gamborg C Thorsen BJ. Consumers’ preferences for bread: transgenic, cisgenic, organic or pesticide-free? J Agric Econ. (2018) 69:121–41. doi: 10.1111/1477-9552.12225

[25] Gizaw Z. Public health risks related to food safety issues in the food market: a systematic literature review. Environ Health Prev Med. (2019) 24:1–21. doi: 10.1186/s12199-019-0825-5 31785611 PMC6885314

[26] Krimsky S. GMOs decoded: A Skeptic's view of genetically modified foods. New York: MIT Press (2019).

[27] Ozkok G. Genetically modified foods and the probable risks on human health. Int J Nutr Food Sci. (2015) 4:356–63. doi: 10.11648/j.ijnfs.20150403.23

[28] Bernstein JA Bernstein LI Bucchini L Goldman LR Hamilton RG Lehrer S. Clinical and laboratory investigation of allergy to genetically modified foods. Environ Health Perspect. (2003) 111:1114–21. doi: 10.1289/ehp.5811 12826483 PMC1241560

[29] Dona A Arvanitoyannis IS. Health risk of genetically modified foods. Crit Rev Food Sci Nutr. (2009) 49:164–75. doi: 10.1080/10408390701855993 18989835

[30] Philips JG Martin-Avila E Robold AV. Horizontal gene transfer from genetically modified plants-regulatory considerations. Front Bioeng Biotechnol. (2022) 10:971402. doi: 10.3389/fbioe.2022.971402 36118580 PMC9471246

[31] Nawaz MA Mesnage R Tsatsakis AM Golokhvast KS Yang SH Antoniou MN. Addressing concerns over the fate of DNA derived from genetically modified food in the human body: A review. Food Chem Toxicol. (2019) 124:423–30. doi: 10.1016/j.fct.2018.12.030 30580028

[32] Kosicka-Gębska M Gębski J. Oczekiwania i obawy związane z wprowadzaniem do obrotu produktów i żywności pochodzących z genetycznych modyfikacji. Problemy Rolnictwa Światowego. Zeszyty Naukowe SGGW w Warszawie. (2009) 9:65–76. (in Polish)

[33] Chen MF Li HL. The consumer's attitude toward genetically modified foods in Taiwan. Food Qual Prefer. (2007) 18:662–74. doi: 10.1016/j.foodqual.2006.10.002

[34] Streiffer R Hedemann T. The political import of intrinsic objections to genetically engineered food. J Agric Environ Ethics. (2005) 18:191–10. doi: 10.1007/s10806-005-0633-3

[35] Rihn A Hayk K Xuan W. Perceived subjective versus objective knowledge: consumer valuation of genetically modified certification on food producing plants. PLoS One. (2021) 16:e0255406. doi: 10.1371/journal.pone.0255406 34411110 PMC8376035

[36] Sikora D Piotr R. olicy Issues in Genetically Modified Crops. In: Public acceptance of GM foods: a global perspective. In: Public acceptance of GM foods: a global perspective. (2021). Singh P Borthakur A Singh AA Kumar A Singh K. editors. Academic Press. 293–15.

[37] Jiang S Wei F. Misinformation and disinformation in science: examining the social diffusion of rumours about GMOs. Cult Sci. (2019) 2:327–40. doi: 10.1177/209660831900200407

[38] Shtulman A Share I Silber-Marker R Landrum AR. OMG GMO! Parent-child conversations about genetically modified foods. Cogn Dev. (2020) 55:100895. doi: 10.1016/j.cogdev.2020.100895

[39] Moerbeek H Casimir G. Gender differences in consumers' acceptance of genetically modified foods. Int J Consum Stud. (2005) 29:308–18. doi: 10.1111/j.1470-6431.2005.00441.x

[40] Moon M. Balasubramanian S.K. Public attitudes toward agrobiotechnology: the mediating role of risk perceptions on the impact of trust, awareness, and outrage. Rev Agric Econ. (2004) 26:186–08. doi: 10.1111/j.1467-9353.2004.00170.x

[41] Vilella-Vila M Costa-Font J Mossialos E. Mossialos consumers involvement and acceptance of biotechnology in the European Union: a specific focus on Spain and the UK. Int J Consum Stud. (2005) 29:108–18. doi: 10.1111/j.1470-6431.2004.00425.x

[42] Costa-Font M Gil JM Traill WB. Consumer acceptance, valuation of and attitudes towards genetically modified food: review and implications for food policy. Food Policy. (2008) 33:99–11. doi: 10.1016/j.foodpol.2007.07.002

[43] Hasell A Stroud NJ. The differential effects of knowledge on perceptions of genetically modified food safety. Int J Public Opin Res. (2020) 32:111–31. doi: 10.1093/ijpor/edz020

[44] Diamond E Thomas B Frederick M. Does providing scientific information affect climate change and GMO policy preferences of the mass public? Insights from survey experiments in Germany and the United States. Environ Polit. 29:1199–218. doi: 10.1080/09644016.2020.1740547

[45] Frewer L Jesper L Kettlitz B Scholderer J Beekman V Berdal GK. Societal aspects of genetically modified foods. Food Chem Toxicol. (2004) 42:1181–93. doi: 10.1016/j.fct.2004.02.002 15123386

[46] Lindberg S Peters DJ Cummings CL. Gene-edited food adoption intentions and institutional Trust in the United States: benefits, acceptance, and labeling. Rural Sociol. (2023) 88:392–25. doi: 10.1111/ruso.12480

[47] Pechar E Bernauer T Frederick M. Beyond political ideology: the impact of attitudes towards government and corporations on trust in science. Sci Commun. (2018) 40:291–13. doi: 10.1177/1075547018763970

[48] Haspel T (2013). Genetically modified foods: what is and isn't true The Washington Post Avaialble at: http://www.washingtonpost.com/lifestyle/food/genetically-modified-foods-what-is-and-isnt-true/2013/10/15/40e4fd58-3132-11e3-8627-c5d7de0a046b_story.html. Accessed March 20 2023

[49] Mintz K. Arguments and actors in recent debates over US genetically modified organisms (GMOs). J Environ Stud Sci. (2017) 7:1–9. doi: 10.1007/s13412-016-0371-z

[50] Champion VL Skinner CS. The health belief model. Health Behav Health educ Theory Res Pract. (2008) 4:45–65.

[51] Bouis HE. The potential of genetically modified food crops to improve human nutrition in developing countries. Transg Poor. (2013) 43:79–96. doi: 10.1080/00220380601055585

[52] Shetty MJ Chandan K Krishna HC Aparna GS. Genetically modified crops: an overview. J Pharmacog Phytochem. (2018) 7:2405–10.

[53] Boudreau G. Behavioural change in environmental education. J Environ Sci Public Health. (2010) 1:120–33.

[54] Hungerford HR Volk TL. Changing learner behavior through environmental education. J Environ Educ. (1990) 21:8–21. doi: 10.1080/00958964.1990.10753743

[55] Sociologický ústav (Akademie věd ČR). Centrum pro výzkum veřejného mínění Potraviny 2021 [datový soubor] (online) (2022) Ver. 1.0. Praha: Český sociálněvědní datový archiv. Cited 6.6.2023

[56] Herman RA Fedorova M Storer NP. Will following the regulatory script for GMOs promote public acceptance of gene-edited crops? Trends Biotechnol. (2019) 37:1272–3. doi: 10.1016/j.tibtech.2019.06.007 31307666

[57] Vindigni G Peri I Consentino F Selvaggi R Spina D. Exploring consumers' attitudes towards food products derived by new plant breeding techniques. Sustainability. (2022) 14:5995. doi: 10.3390/su14105995

[58] Woźniak-Gientka E Tyczewska A Twardowski T. Public opinion on biotechnology and genetic engineering in the European Union: polish consumer study. Biotechnologia. (2022) 103:185–01. doi: 10.5114/bta.2022.116212 36606075 PMC9642953

[59] Clancy KA Clancy B. Growing monstrous organisms: the construction of anti-GMO visual rhetoric through digital media. Crit Stud Media Commun. (2016) 33:279–92. doi: 10.1080/15295036.2016.1193670

[60] Dayé C Spök A Allan AC Yamaguchi T Sprink T. Social acceptability of cisgenic plants: public perception, consumer preferences, and legal regulation. Cisgenic Crops Safe Legal Soc Issues:43–75. doi: 10.1007/978-3-031-10721-4_3

