# Supplementary material for: The moderating role of perceived health risks on the acceptance of genetically modified food
Source: Front Public Health. 2024 Jan 25;11:1275287. doi: 10.3389/fpubh.2023.1275287 (PMC10851272; doi:10.3389/fpubh.2023.1275287)
Supplement: Supplementary file 1 [file Table_1.docx]

# Appendix A1. The results presented in Table 8 in bulleted form

**Health risks**

- The subjective assessment of own health condition proved unrelated to GM attitudes and actions.
- The more the respondents believe that GMF is safe, (1) the more they would purchase foods with GM ingredients and (2) the less they consider GMF immoral; (3) the more likely they would try GMF.
- The more the respondents believe that GMF can change their DNA, (1) the less likely they would buy food with a GM ingredient, (2) the more they consider GMF immoral.
- The more the respondents believe that GMF can endanger their health, (1) the less likely they would buy food with a GM ingredient, (2) the more they consider GMF immoral; (3) the less likely they are to try food with GM ingredients.

**Environmental concerns**

- The more important the effect of food production on the environment, the less likely they would try GMF.
- The more the respondent engage in waste management, the more likely they are to try GMF.

**Information and interest**

- The more the respondent is informed about GMF, (1) the more likely he would buy food with a GM ingredient, (2) the less he believes that GMF is morally unacceptable.
- Interest in GMF positively predicts (1) GMF considered immoral: the more the respondents are interested in GMF, the more they consider GMF immoral;
- The more the respondents believe that the effects of GM are scientifically investigated, (1) the more likely they would buy food with a GM ingredient.

**Socio-demographics and other controls**

- The bigger the city, the more people are willing to try GMF
- The more the respondent is satisfied in his life, the more he is willing to try GMF
