# Supplementary material for: The moderating role of perceived health risks on the acceptance of genetically modified food
Source: Front Public Health. 2024 Jan 25;11:1275287. doi: 10.3389/fpubh.2023.1275287 (PMC10851272; doi:10.3389/fpubh.2023.1275287)
Supplement: Supplementary file 3 [file Table_3.docx]

## The questionnaire. The part used in the paper, English translation

## GMF attitudes, information, perceived effects

"We will now focus on the topic of genetically modified, or modified organisms, i.e. crops from which, among other things, the foods we consume are produced."

PL.20 “Have you heard about genetically modified crops?

- No, you haven't heard of genetic modification of crops. … 1
- you have heard about genetic modification of crops, but you do not know what it refers to,..... 2
- you have heard about genetic modification of crops and you roughly know what it refers to,............ 3
- you have heard about the genetic modification of crops and you know very well what it refers to."............. 4

PL.21 "Are you personally interested in genetically modified crops?

- You are definitely interested, 1
- rather you are interested in 2
- rather you are not interested, 3
- are you definitely not interested 4
- DON'T KNOW 9

PL.22 "Do you have enough or insufficient information about genetically modified crops?"

- Definitely enough, ........................................ 1
- rather enough, .............................................. 2
- rather a lack, .............................................. 3
- definitely a lack?” .............................................. 4
- DON'T KNOW ................................................ 9

PL.23 "When buying food, how often do you check on the label or description of the food whether it contains ingredients from genetically modified crops?"

- Always, ................................................ 1
- often, ................................................ 2
- rarely, ................................................ 3
- never." ............................................... 4
- DON'T KNOW ................................................ 9

PL.91 “If you had the opportunity, would you taste an approved and verified food from genetically modified crops?

- He must have tasted it................................................... 1
- rather tasted................................................... 2
- rather he didn't taste it, ........................................ 3
- he certainly didn't taste it."................................................ 4
- DON'T KNOW ................................................ 9

PL.92 "Can or cannot, in your opinion, foods from genetically modified crops endanger your health?"

- They certainly can,................................................ 1
- rather they can,................................................ 2
- rather they can't, .............................................. 3
- they certainly can't."................................................ 4
- DON'T KNOW ................................................ 9

PL.93 “How many of the foods you normally eat do you think contain ingredients from genetically modified crops?

- None or almost none,............................................ 1
- rather a minority,................................................ 2
- about half,................................................ 3
- rather most,................................................ 4
- all or almost all."................................................ 5
- DON'T KNOW ................................................ 9

INSTRUCTION: GIVE THE CARD PL.94 TO THE RESPONDENT

CARD PL.94 “To what extent do you agree or disagree with the following statements?

- STRONGLY AGREE 1
- HE RATHER AGREES 2
- NEITHER AGREE NOR DISAGREE 3
- RATHER DISAGREE 4
- STRONGLY DISAGREE 5
- HE DOESN'T KNOW 9

a) Eating food from genetically modified crops is safe. 1 2 3 4 5 9

b) The effects of food from genetically modified crops on human health are scientifically investigated. 1 2 3 4 5 9

c) If you discovered that you had a food item in your shopping cart containing an ingredient from genetically modified crops, you would still buy it. 1 2 3 4 5 9

d) Food containing ingredients from genetically modified crops should have this information in the description or on the label. 1 2 3 4 5 9

e) Genetically modifying crops is morally unacceptable.” 1 2 3 4 5 9

PL.95 “Can or cannot, in your opinion, eating food from genetically modified crops change your DNA?

- It certainly can, .............................................. 1
- rather can, .............................................. 2
- rather he can't, .............................................. 3
- surely he can't."................................................ 4
- DON'T KNOW ................................................9

## Environmental concerns

PL.56 "When buying food, is it important or unimportant to you what the impact of its production is on the environment?"

- Definitely important, ................................................ 1
- rather important, ................................................ 2
- rather unimportant, ................................................ 3
- absolutely unimportant?”................................................ 4
- DON'T KNOW ................................................ 9

INSTRUCTION: HAND THE PL.55 CARD TO THE RESPONDENT

PL.55 “Regarding you or your household, how often do you do the following activities?

- NEVER 1
- RARELY 2
- SOMETIMES 3
- OFTEN 4
- ALWAYS 5
- HE DOESN'T KNOW 9

a) You carry your own bag for shopping. 1 2 3 4 5 9

b) You buy fruit and vegetables in so-called "endless bags" 1 2 3 4 5 9

c) You use your own reusable drinking bottle 1 2 3 4 5 9

d) You use environmentally friendly products for household activities (e.g. cleaning, laundry). 1 2 3 4 5 9

e) When purchasing, you prefer food produced in the Czech Republic. 1 2 3 4 5 9

f) You prepare food in your own boxes. 1 2 3 4 5 9

g) You avoid disposable plastic products (e.g. cutlery, straws). 1 2 3 4 5 9

h) You limit driving for reasons of environmental protection. 1 2 3 4 5 9

i) You save energy and water for the sake of environmental protection. 1 2 3 4 5 9

j) You sort ordinary waste. 1 2 3 4 5 9

k) You compost." 1 2 3 4 5 9

## The importance of food characteristics when purchasing

PL.51 "Please rank the following options according to how important they are to you when shopping for food. Please go from most to least important, with 1 being most important and 6 being least important.” DON'T KNOW = 9

- Origin
- packaging material
- price
- Ingredients
- package size
- method of breeding/growing food or its raw materials

### The importance of food and food habits

PL.80 "Is it important or unimportant to you how you eat?"

- Definitely important, ................................................ 1
- rather important, ................................................ 2
- rather unimportant, ................................................ 3
- absolutely unimportant?”................................................ 4
- DON'T KNOW ................................................9

PL.81 “How many meals a day do you usually eat?

- One meal,................................................... 1
- two meals................................................... 2
- three meals................................................... 3
- four meals................................................... 4
- five meals,................................................... 5
- more than five meals a day." ............................6
- DON'T KNOW................................................ 9

PL.41 “How often do you buy groceries?

- Daily, ............................................... 1
- several times a week, ................................................ 2
- 1x a week, ............................................... 3
- 1x in 14 days, ............................................ 4
- less often than once in 14 days." ......................................... 5
- DON'T KNOW ................................................ 9

## Socio-economic characteristics of the respondents and other

IDE.8 THE RESPONDENT IS:

- MAN = 1
- WOMAN = 2

IDE.2 "How old are you?" (write a number)

INSTRUCTION: GIVE THE IDE CARD TO THE QUESTIONER.6b

IDE.6b "What is your highest completed education?"

- NO ANSWER 0
- unfinished basic 1
- completed basic 2
- apprenticeship 3
- high school without matriculation 4
- secondary vocational school with matriculation 5
- secondary general with 6th matriculation
- higher professional 7
- university bachelor 8
- university master's, engineering 9
- postgraduate education, scientific training 10

OZ.4 "How would you rate your state of health?" As

- very good, ................................................ 1
- good, ................................................ 2
- average, ................................................ 3
- not good, ................................................ 4
- very bad." ................................................ 5
- DON'T KNOW ................................................9

IDE.1 "You consider the standard of living of your household

- for very good, .............................................. 1
- for rather good, .............................................. 2
- neither good nor bad, ............................................ 3
- for a rather bad one, ........................................ 4
- or for a very bad one?” ............................................ 5
- DON'T KNOW ................................................ 9

OV.1 "How satisfied are you with your life overall?"

- You are very satisfied, ........................................ 1
- rather satisfied, .............................................. 2
- neither satisfied nor dissatisfied, ............................................ 3
- rather dissatisfied, ................................................ 4
- very dissatisfied?” .............................................. 5
- DON'T KNOW ................................................ 9

IDE.19 "Looking at the IDE.19 card, how would you indicate where you live?"

IDE 19 CARD:

- NO ANSWER 0
- big city 1
- a suburb of a large city or a residence in the immediate vicinity 2
- medium-sized city 3
- small town 4
- big village 5
- small village, settlement, solitude 6
- other type of seat 7
- DOESN'T KNOW 9

IDE.7 "Which church or religious community do you belong to?"

- NO ANSWER 0
- Roman Catholic 1
- Protestant 2
- orthodox, orthodox 3
- Jewish 4
- Muslim 5
- other 6
- not a believer 7
- DOESN'T KNOW 9
