# Supplementary material for: The moderating role of perceived health risks on the acceptance of genetically modified food
Source: Front Public Health. 2024 Jan 25;11:1275287. doi: 10.3389/fpubh.2023.1275287 (PMC10851272; doi:10.3389/fpubh.2023.1275287)
Supplement: Supplementary file 4 [file Table_4.docx]

**The SPSS syntax**

[DataSet6] C:\Users\cabelkova\PRACE 2023\my papers\potraviny\CSDA00294_F1 data potraviny program.sav

Categories 0, 7, 997 and other, defining missing information were defined as missing

**Trasformation of variables**

Category 9 (undecided} is joined with category 3 (no opinion)

COMPUTE PL_94C_1=ANY(PL_94C,1)*1+ANY(PL_94C,2)*2+ANY(PL_94C,3,

9)*3+ANY(PL_94C,4)*4++ANY(PL_94C,5)*5.

EXECUTE.

The other variables were transformed similarly

**Descriptive statistics:**

FREQUENCIES VARIABLES=PL_20 PL_21 PL_23 PL_91 PL_92 PL_93 PL_94A PL_94B PL_94C PL_94D PL_94E

PL_22_1 PL_92_1 PL_94B1 PL_94C1 PL_94D_1 PL_94А1 PL_94Е_1 PL_90 PL_95 PL_95_1 PL_51a PL_51b PL_51c

PL_51d PL_51e PL_51f PL_56 PL_55A PL_55B PL_55C PL_55D PL_55E PL_55F PL_55G PL_55H PL_55I PL_55J

PL_55K PL_81 PL_80

/ORDER=ANALYSIS.

Note: the variables _1 are those, where the categories of no opinion were joined with the group Undecided

Descriptive statistics for continuous variables: ex. Аge (IDE_2]:

DESCRIPTIVES VARIABLES=IDE_2

/STATISTICS=MEAN STDDEV MIN MAX.

**Principal component analysis with saved factors scores (table 7)**

FACTOR

/VARIABLES PL_55A PL_55B PL_55C PL_55D PL_55E PL_55F PL_55G PL_55H PL_55I PL_55J PL_55K

/MISSING LISTWISE

/ANALYSIS PL_55A PL_55B PL_55C PL_55D PL_55E PL_55F PL_55G PL_55H PL_55I PL_55J PL_55K

/PRINT UNIVARIATE INITIAL CORRELATION SIG KMO EXTRACTION ROTATION FSCORE

/FORMAT SORT

/PLOT EIGEN ROTATION

/CRITERIA MINEIGEN(1) ITERATE(25)

/EXTRACTION PC

/CRITERIA KAISER ITERATE(25)

/ROTATION VARIMAX

/SAVE REG(ALL)

/METHOD=CORRELATION.

**Ordinal regression analyses, Table 8.**

GET

FILE='C:\Users\cabelkova\PRACE 2023\my papers\potraviny\CSDA00294_F1 data potraviny program.sav'.

DATASET NAME DataSet1 WINDOW=FRONT.

PLUM PL_94C1 BY IDE_8 NonBeliever WITH PL_20 PL_21 PL_22_1 OZ_4 PL_94A PL_94B1 PL_95_1 PL_92_1

PL_56 EnviroRecycling EnviroReduceWaste EnviroSaveResourse PL_51a PL_51b PL_51c PL_51d PL_51e PL_80

PL_81 PL_41 IDE_2 t_VZD IDE_19 IDE_1 OV_1

/CRITERIA=CIN(95) DELTA(0) LCONVERGE(0) MXITER(100) MXSTEP(5) PCONVERGE(1.0E-6) SINGULAR(1.0E-8)

/LINK=LOGIT

/PRINT=FIT PARAMETER SUMMARY.

PLUM PL_94А1 BY IDE_8 NonBeliever WITH PL_20 PL_21 PL_22_1 OZ_4 PL_94A PL_94B1 PL_95_1 PL_92_1

PL_56 EnviroRecycling EnviroReduceWaste EnviroSaveResourse PL_51a PL_51b PL_51c PL_51d PL_51e PL_80

PL_81 PL_41 IDE_2 t_VZD IDE_19 IDE_1 OV_1

/CRITERIA=CIN(95) DELTA(0) LCONVERGE(0) MXITER(100) MXSTEP(5) PCONVERGE(1.0E-6) SINGULAR(1.0E-8)

/LINK=LOGIT

/PRINT=FIT PARAMETER SUMMARY.

PLUM PL_94Е_1 BY IDE_8 NonBeliever WITH PL_20 PL_21 PL_22_1 OZ_4 PL_94A PL_94B1 PL_95_1 PL_92_1

PL_56 EnviroRecycling EnviroReduceWaste EnviroSaveResourse PL_51a PL_51b PL_51c PL_51d PL_51e PL_80

PL_81 PL_41 IDE_2 t_VZD IDE_19 IDE_1 OV_1

/CRITERIA=CIN(95) DELTA(0) LCONVERGE(0) MXITER(100) MXSTEP(5) PCONVERGE(1.0E-6) SINGULAR(1.0E-8)

/LINK=LOGIT

/PRINT=FIT PARAMETER SUMMARY.

**Ordinal regression analyses, Table 9.**

PLUM PL_94А1 BY IDE_8 NonBeliever WITH PL_20 PL_21 PL_22_1 OZ_4 PL_56 EnviroRecycling

EnviroReduceWaste EnviroSaveResourse PL_51a PL_51b PL_51c PL_51d PL_51e PL_80 PL_81 PL_41 IDE_2

t_VZD IDE_19 IDE_1 OV_1

/CRITERIA=CIN(95) DELTA(0) LCONVERGE(0) MXITER(100) MXSTEP(5) PCONVERGE(1.0E-6) SINGULAR(1.0E-8)

/LINK=LOGIT

/PRINT=FIT PARAMETER SUMMARY.

PLUM PL_94Е_1 BY IDE_8 NonBeliever WITH PL_20 PL_21 PL_22_1 OZ_4 PL_56 EnviroRecycling

EnviroReduceWaste EnviroSaveResourse PL_51a PL_51b PL_51c PL_51d PL_51e PL_80 PL_81 PL_41 IDE_2

t_VZD IDE_19 IDE_1 OV_1

/CRITERIA=CIN(95) DELTA(0) LCONVERGE(0) MXITER(100) MXSTEP(5) PCONVERGE(1.0E-6) SINGULAR(1.0E-8)

/LINK=LOGIT

/PRINT=FIT PARAMETER SUMMARY.

PLUM PL_94C1 BY IDE_8 NonBeliever WITH PL_20 PL_21 PL_22_1 OZ_4 PL_56 EnviroRecycling

EnviroReduceWaste EnviroSaveResourse PL_51a PL_51b PL_51c PL_51d PL_51e PL_80 PL_81 PL_41 IDE_2

t_VZD IDE_19 IDE_1 OV_1

/CRITERIA=CIN(95) DELTA(0) LCONVERGE(0) MXITER(100) MXSTEP(5) PCONVERGE(1.0E-6) SINGULAR(1.0E-8)

/LINK=LOGIT

/PRINT=FIT PARAMETER SUMMARY.
